# Therapeutic drug monitoring practices of anti-infectives: An Asia-wide cross-sectional survey

**DOI:** 10.3389/fphar.2022.992354

**Published:** 2022-10-10

**Authors:** Jingjing Hou, Debbie Marriott, Dario Cattaneo, Sophie Stocker, Jana Stojanova, Jan-Willem Alffenaar, Chenlin Xiao, Yichang Zhao, Hui Gong, Miao Yan

**Affiliations:** ^1^ Department of Pharmacy, The Second Xiangya Hospital, Central South University, Changsha, China; ^2^ International Research Center for Precision Medicine, Transformative Technology and Software Services, Changsha, China.; ^3^ Department of Microbiology and Infectious Diseases, St. Vincent’s Hospital, Sydney, NSW, Australia; ^4^ Unit of Clinical Pharmacology, ASST FBF Sacco University Hospital, Milan, Italy; ^5^ School of Pharmacy, Faculty of Medicine and Health, The University of Sydney, Sydney, NSW, Australia; ^6^ UNSW Sydney, St Vincent’s Clinical School, Sydney, Australia; ^7^ Department of Clinical Pharmacology and Toxicology, St Vincent’s Hospital Sydney, Sydney, NSW, Australia; ^8^ Westmead Hospital, Sydney, NSW, Australia; ^9^ Sydney Institute for Infectious Diseases, University of Sydney, Sydney, NSW, Australia

**Keywords:** therapeutic drug monitoring, anti-infective, interpretation, clinical practice, questionnaire

## Abstract

**Objectives:** The current practice of therapeutic drug monitoring (TDM) in Asia is poorly documented. Our aim was to capture and describe TDM services delivered in hospitals across Asia, including aspects such as assay availability, interpretation of results and clinical decision-making.

**Methods:** An online survey about anti-infective TDM practices, available in English and involving 50 questions, was promoted to people involved in TDM in Asia. The survey was open for responses from September to November 2021.

**Results:** Of 207 responses from participants working in 14 Asian countries, 150 responses from 10 countries could be included. TDM services are available for many anti-infectives, providing assays based on chromatographic assays (100.0%) or immunoassays (39.3%). Clinicians (82.6%) and pharmacists (86.8%) were responsible for ordering and interpreting TDM. Most services provided reference targets and dose recommendations. Interpretative support was available to a varying degree. Assay results were available and clinical decision-making could be completed within 24 h in most hospitals (87.9% and 88.9% respectively). As the turnaround time of assay results decreased, the proportion of clinical decision-making completed within 8 h increased. Barriers to implementation of TDM included lack of funding or equipment (71.1%), lack of clinician interest or cooperation (47.0%), and lack of expertise (42.3%). Lack of expertise was the primary barrier for using precision dosing software (50.5%).

**Conclusion:** There are significant differences and challenges in the development and practice of anti-infective TDM in Asian countries.

## Introduction

Antibiotic resistance is increasing globally and few new antimicrobial agents are entering the market (The Review on Antimicrobial Resistance, 2016). Optimal, timely and appropriate anti-infective therapy is an important public health issue to minimise the development of antimicrobial resistance in available agents. Therapeutic drug monitoring (TDM) represents a strategy to personalize anti-infective therapy, leading to improved outcomes and avoiding resistance, particularly in the hospital setting where the development of resistance is of particular concern (Eliasson et al., 2013). TDM involves the measurement of the drug concentrations in blood or other validated specimen, interpretation of the result within the clinical context, and adjustment of the dose to achieve concentrations within the therapeutic range, thereby improving therapeutic efficacy and reducing the risk of concentration-dependent adverse effects (Abdul-Aziz et al., 2020; Vena et al., 2020).

TDM practices for anti-infectives are well described in Europe and North America (Jorgensen et al., 2021; Lanckohr et al., 2021), however, the pattern and extent of TDM implementation in Asian countries is poorly documented. TDM may be especially important in the Asia region given racial differences in key pharmacokinetic process. In a randomized 2-way crossover study, Koreans showed area under the concentration-time curve (AUC) for erythromycin that was 1.66 times higher than those of Caucasians (Yu et al., 2001). Numerous other examples of bioavailability and clearance that result in differential exposure in people of Asian descent have been described (Chen, 2006; Zhou et al., 2020; Zhou et al., 2021). Given vast differences in economic development and healthcare infrastructure across Asia, alongside cultural differences, it is anticipated that TDM practices may vary substantially compared to Europe and North America (Younger, 2016; Agustina et al., 2019; Haenssgen et al., 2019). Despite a growing body of literature regarding antimicrobial TDM, there is a paucity of reports concerning TDM practices in Asia. We therefore conducted a survey to evaluate current antimicrobial TDM practices in the region, including aspects such as assay availability, interpretation of results and clinical decision-making.

## Materials and methods

### Ethical approval

The survey involved procedural questions that were not of a sensitive nature. No information that could identify participants was collected. People were contacted by email and were free to choose whether they wished to participate. Responding to the survey was considered implied consent of the respondent. No compensation was offered to participants. Despite the nature of the questionnaire and the negligible-risk of the study, we sought ethical approval to the Ethics Committee of The Second XiangYa Hospital of Central South University with an approved [(2022) Ethical Review [C.R] No. (K049)] to conduct the survey.

### Study design

A cross-sectional study was conducted by an online survey to assess TDM practices for anti-infective agents in Asia. A multidisciplinary group of medical and pharmacist anti-infectives specialists and TDM laboratory specialists developed a four-part survey with 50 questions and was based on previously published surveys (Tabah et al., 2015; Chen et al., 2018; He et al., 2020; Imani et al., 2020; Liu et al., 2021; Wicha et al., 2021). The survey was available in English and took about 10 min to complete (Supplementary material). Part one (12 questions) captured the demographic details of respondents and their healthcare institutions. Parts two and three captured information on availability of TDM for anti-infective agents, assay methods, and sampling (6 questions, respectively). The last part (26 questions) focused on institutional TDM practice. Clinical vignettes were used to evaluate institutional TDM practice in general and clinical decision-making for vancomycin and voriconazole, the most common anti-infectives managed by TDM. Specifically, this section focused on interpretation, clinical response and decision-making based on TDM results, including defining targets and critical values, the combination with MIC values and the individualized regimen of special populations or specific situation (for example, the next round of ordering TDM). To accurately capture differences in TDM practice, specific questions used branching logic, or remained open-ended with response options not mutually exclusive. The option to respond in free-text was also offered. The survey was pilot tested by nine experts in the field of TDM (seven Chinese, one Thai and one Australian) and was hosted on the Research Electronic Data Capture (REDCap) web application.

### Study population

An email invitation to complete the survey was distributed to members of the International Association of Therapeutic Drug Monitoring & Clinical Toxicology (IATDMCT), registrants of the IATDMCT Asia-Pacific Regional Meeting and Division of Therapeutic Drug Monitoring and Chinese Pharmacological Society (China-TDM). The survey was also promoted by the authors within their professional networks.

### Study procedure

The survey was conducted between September and November 2021. Following an initial invitation, three e-mail reminders were sent at 2, 3, 5 and 6 weeks. The survey was closed at 9 weeks as no further responses were captured. No identifying information was collected; a question to indicate the name of the institution where respondents work was voluntary and aimed to identify multiple responses from a single site. We did not measure distribution reach thus the response rate was not determined.

### Statistical analysis

When the field “other” provided the option to enter free-text, answers were coded and sorted where possible. Only completed responses were analyzed, and the number of answers to each question were counted. For descriptive statistics used to summarize survey responses, categorical variables were expressed in number and/or percentage. For inferential statistics, nonparametric Spearman’s rank correlation coefficient and χ2 linear association test was used to study the relationship between two processes of TDM, as appropriate. Data analyses were performed using IBM SPSS Statistics version 25 (IBM, New York, 363NY). Figures were created using GraphPad Prism version 8 (San Diego, CA, United States) and OriginPro 2021b 9.8.5.212 (Learning Edition) (2012 OriginLab Corporation, Northampton, MA 01060, United States). Two-sided tests with a *p*-value of <0.05 was considered statistically significant.

## Results

### Respondent and institutional characteristics

**FIGURE 1:**
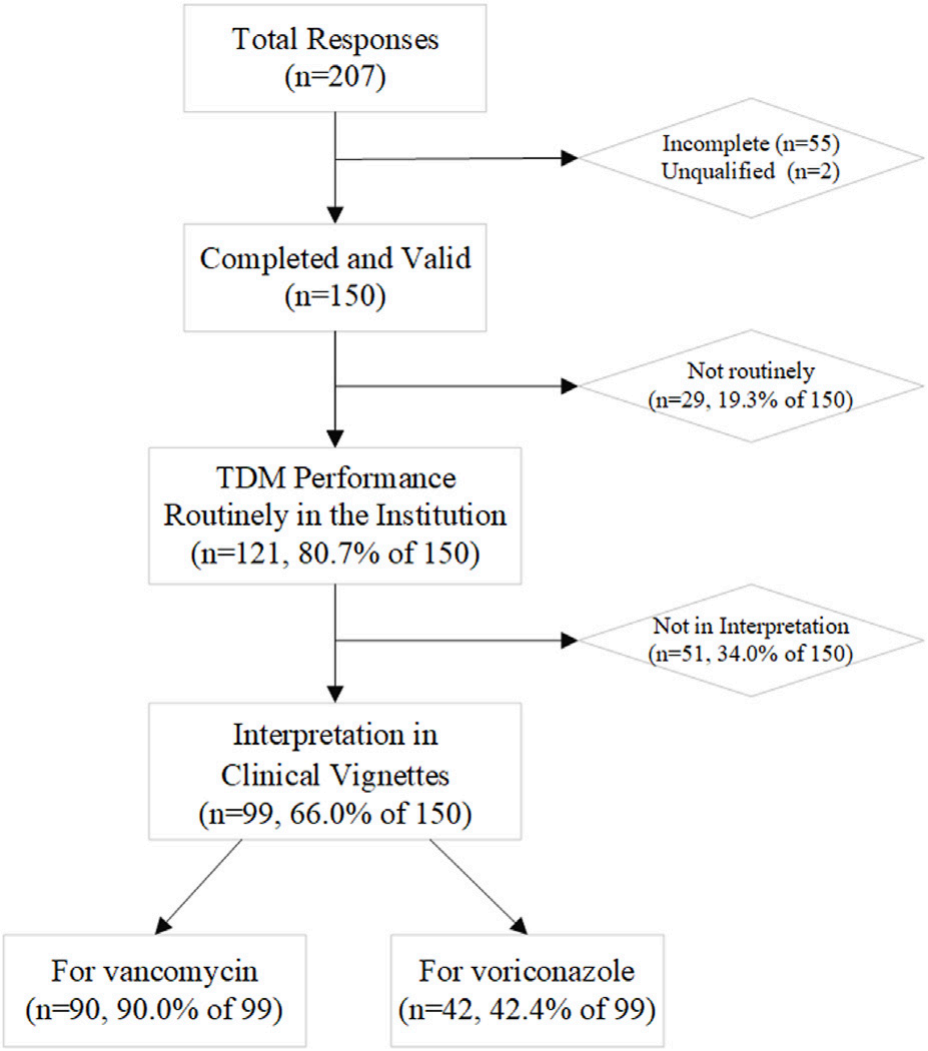
The flow chart shows the feedback of different sections.

Two hundred and seven health professionals from 14 Asian countries responded; 150 respondents from 10 countries were included in description and statistical analysis: two were deemed ineligible and 55 were incomplete. TDM services were available at the institutions of 121 respondents (80.7%) (Figure 1), which has been increasing over time (Supplementary Figure S1).

Respondents were predominantly from China, Malaysia and India (36.0%, 27.3% and 20.7% respectively, 84.0% in total; Table 1). Most respondents were pharmacists (60.0%), with 5–10 years (38.0%) or over 10 years (15.7%) experience in delivering TDM (Table 2). Most respondents indicated they had institutional guidelines in place for TDM, but 14.0% indicated poor guideline compliance. Most laboratories participate in annual or more frequent external quality assessment (55.4%), with either domestic (34.7%) or international (15.3%; Table 2) schemes (e.g., United Kingdom National External Quality Assessment Scheme, UKNEQAS).

**TABLE 1:** Number of respondents by country.

Country	n (N = 150^a^)	%
China	54	36.0
India	31	20.7
Indonesia	7	4.7
Japan	4	2.7
Kuwait	1	0.7
Malaysia	41	27.3
Nepal	1	0.7
Singapore	5	3.3
Thailand	2	1.3
Vietnam	4	2.7

a A total of 150 respondents finished the survey. Besides, there was feedback from Afghanistan, Philippines, the United Arab Emirates and Turkey (*n* = 4, 1, 1, 1, respectively), but it was not included due to incomplete.

**TABLE 2:** Respondent and hospital characteristics.

	n	%
Respondent		
Position	N = 150	
Clinical pharmacist	90	60.0
Academic position or others	33	22.0
Clinical pharmacologist	31	20.7
Laboratory staff	13	8.7
Clinician	6	4.0
Experience in TDM (years)	N = 121^a^	
<5	45	37.2
5–10	46	38.0
>10	19	15.7
Not yet engaged	11	9.1
Hospital and Laboratory		
SOP for sample collection	N = 150	
Consistent with guidelines	72	48.0
Internal only	57	38.0
Poor compliance	21	14.0
External quality assessment	N = 150	
Annual or more frequent domestic	52	34.7
Annual or more frequent international	23	15.3
Internal only	34	22.7
Unclear	40	26.7
No	15	10.0
Certified	N = 121^a^	
Yes	67	55.4
No	22	18.2
Unclear	32	26.4

a A total of 121 respondents answered this question, because the medical facility they worked for was using TDM, of any drug for clinical care.

TDM, therapeutic drug monitoring; SOP, standard operating procedure.

### Responsibilities and roles for TDM

**FIGURE 2:**
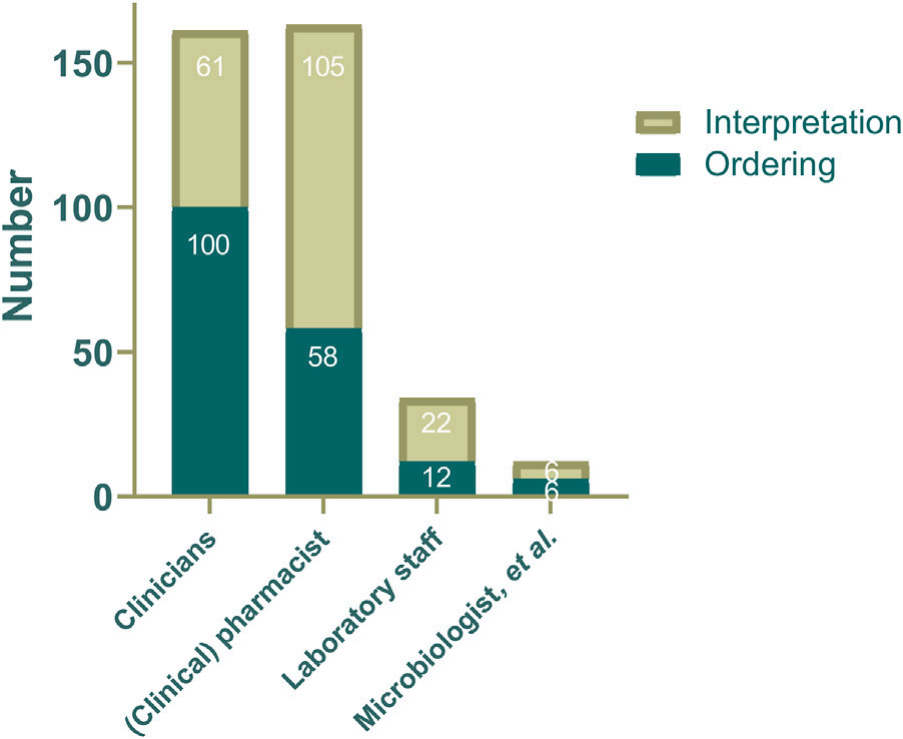
The distribution of professionals responsible for ordering or interpretation anti-infective agents TDM service at each institution (N = 121).

Clinicians were predominantly responsible for ordering TDM for anti-infective agents (82.6%), followed by pharmacists (47.9%), laboratory staff (9.9%) or other professionals (5.0%; Figure 2 and Table 3). Interpretation of TDM results was predominantly performed by pharmacists (86.8%), followed by clinicians (50.4%), laboratory staff (18.2%), microbiologist and other health professionals (5.0%). Pharmacy laboratories were most commonly responsible for governance over TDM services (38.8%), followed by clinical pharmacology laboratories (28.1%) and analytical chemistry laboratories (24.0%; Table 3).

**TABLE 3:** Implementation of TDM service.

	n	%
Range (population and departments)	N = 150	
All and routinely	86	57.3
Partly	35	23.3
Not yet	29	19.3
Division	N = 121^a^	
Pharmacy laboratory	47	38.8
Clinical pharmacology laboratory	34	28.1
Analytical chemical laboratory	29	24.0
Pharmacology or microbiology laboratory	7	5.8
University, private laboratory or other third party	4	3.3
Ordering TDM^b^	N = 121^a^	
Clinician	100	82.6
Clinical pharmacist	58	47.9
Laboratory staff	12	9.9
Microbiologist and other health professionals	6	5.0
Interpretation of TDM results^b^	N = 121^a^	
Clinician	61	50.4
Clinical pharmacist	105	86.8
Laboratory staff	22	18.2
Microbiologist and other health professionals	6	5.0
The number of TDM service for all available drugs (average in the last 2 weeks)	N = 121^a^	
>200	21	17.4
100–199	27	22.3
50–99	18	14.9
20–49	22	18.2
<20	33	27.3
Dose prediction software	21	21.1^c^
Assay used for anti-infectives TDM	N = 150^d^	
HPLC	59	39.3
HPLC/MS-MS or LC/MS-MS	54	36.0
HPLC-UV or LC -UV	14	9.3
UPLC	12	8.0
GC	3	2.0
Chemiluminescence immunoassay	36	24.0
Fluorescence polarization immunoassay	21	14.0
Enzyme linked immunosorbent assay	14	9.3
Enzyme Immunoassay	2	1.4
Point-of-care (e.g., micro-dialysis)	1	0.7
Other smaller scale devices	1	0.7
Unclear	43	28.7

a A total of 121 respondents answered this question, who work in medical facility that use TDM, for clinical care.

b All the matching answers can be selected in this question, resulting in the sum percentage exceeds 100%.

c A total of 99 respondents participated in interpretation.

d A total of 150 respondents answered the question.

TDM, therapeutic drug monitoring; HLA, human leukocyte antigen; DBS, dried blood spot; CSF, cerebrospinal fluid; BALF, bronchoalveolar lavage fluid; HPLC, high performance liquid chromatography; MS, mass spectrum; -MS, tandem mass spectrometry; -UV, tandem ultraviolet and visible spectrophotometry; UPLC, Ultra performance liquid chromatography; GC, gas chromatography.

### Current use of TDM

Regarding assay methods, 39.3% of respondents reported availability of immunoassays at their institution, while 100.0% reported chromatographic methods (Table 3). TDM was most commonly performed for antibacterial drugs (72.7% of respondents, Table 4), as are China, Malaysia, India (92.6% of 54, 97.6% of 41, 48.4% of 31, respectively, Supplementary Table S1) and the other countries (79.2% of 24). While 56.7% reported assay availability for anti-tuberculosis drugs, 33.3% indicated amikacin, and 24.0% reported others other than amikacin; 46.7% of respondents indicated availability of assays for antifungals. Most respondents reported availability of assays for glycopeptides (71.3%), 46.7% for aminoglycosides, 40.0% for voriconazole and 23.3% for *β*-lactams (Table 4). 15.3% respondents did not have any anti-infective assay available at their institutions, most of whom are clinical pharmacologist (7.3%) and academic professionals (4.7%). 3.7% of Chinese respondents and 48.4% of Indian respondents reported no implementation of anti-infectives TDM (*n* = 54, 31, respectively). Forty-one respondents in Malaysia reported that 97.6% practiced at least one anti-infectives TDM, involving four categories of antibacterial drugs and seven anti-tuberculosis drugs, but no antifungal drugs (Supplementary Table S1).

**TABLE 4:** The TDM available anti-infective agents.

	N	% (of 150)
None of any anti-infectives	23	15.3
Antibacterial drugs		
None	26	17.3
Glycopeptides (Vancomycin, Norvancomycin, Teicoplanin, *etc.*)	107	71.3
Aminoglycosides (Gentamicin, Amikacin, Tobramycin, *etc.*)	70	46.7
*β*-lactams (including the carbapenems such as Meropenem.)	35	23.3
Oxazolidinones (e.g., linezolid)	21	14.0
Polypeptide antibiotic (e.g., polymyxin B)	16	11.3
Quinolones (e.g., moxifloxacin, ciprofloxacin)	7	4.7
Sulfonamides (e.g., Sulfamethoxazole)	5	3.3
Tigecycline	2	1.4
Fosfomycin	1	0.7
Unclear	9	6.0
Antifungal drugs		
None	80	53.3
Voriconazole	60	40.0
Posaconazole	20	13.3
Itraconazole	15	10.0
Amphotericin B	11	7.3
Caspofungin	8	5.3
Flucytosine	3	2.0
Isavuconazole	2	1.4
Unclear	1	0.7
Anti-tuberculosis drugs		
None	65	43.3
Isoniazid	22	14.7
Rifampicin	17	11.3
Pyrazinamide	10	6.7
Streptomycin	7	4.7
Ethambutols	7	4.7
Rifabutin	4	2.7
Para-amino salicylic acid	1	0.7
Kanamycin	4	2.7
Linezolid	18	12.0
Ethionamide	1	0.7
Levofloxacin	10	6.7
Moxifloxacin	4	2.7
Bedaqualine	2	1.3
Clofazimine	1	0.7
Delamanid	1	0.7
Imipenem-cilastatin	10	6.7
Amikacin	50	33.3
Meropenem	21	14.0

### Clinical vignettes for interpretation

**FIGURE 3:**
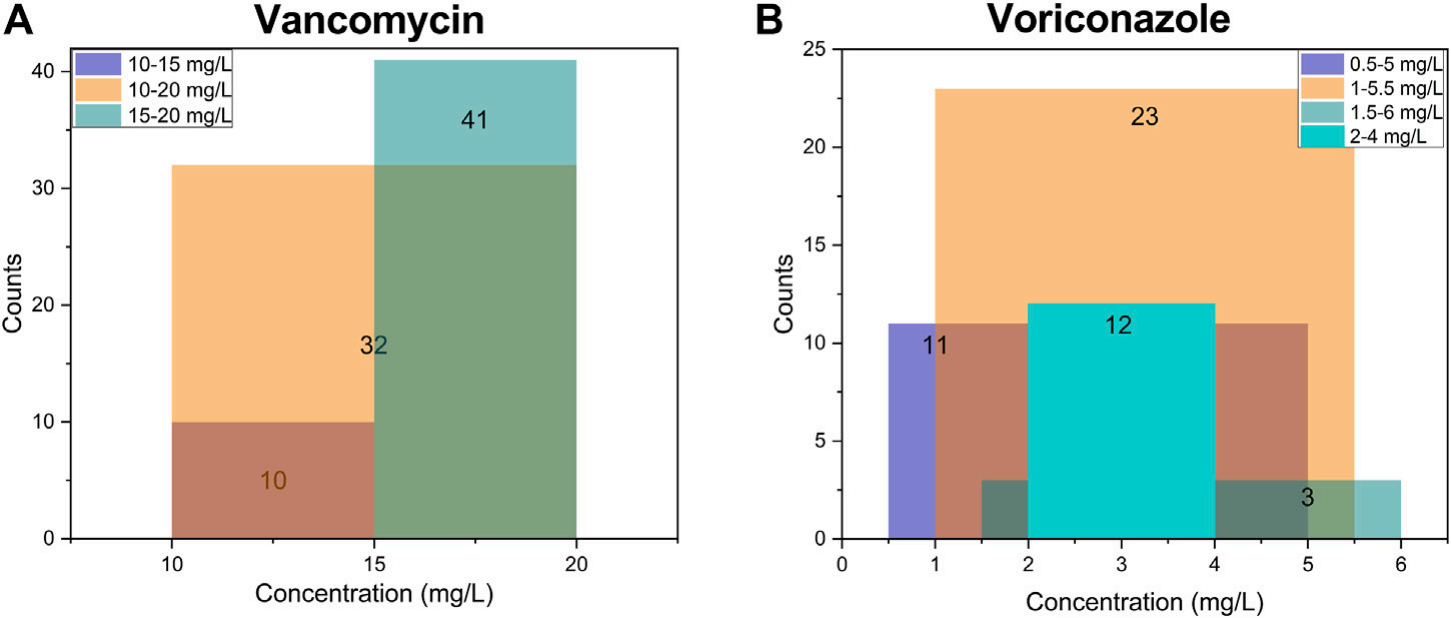
The top three target concentration ranges and their overlap in all institutions (N = 99). **(A)** of vancomycin for adult patients infected with MRSA (N = 90); **(B)** of voriconazole for adult patients (N = 42), and 12 respondents pursuing 2–4 mg/L as the target range considering clinical efficacy and safety in critically ill patients. The numbers on the column represent the respective counts. The color of the overlap will change when the target ranges cross, and the range with the most overlaps means the most common part of the target concentration.

Two-thirds of respondents completed at least one of the clinical vignettes (*n* = 99, 66.0%; Figure 1 and Table 5). Most of those who did not participate in the interpretation were staff in academic or other positions (21, 41.2%) and clinical pharmacist or pharmacist (20, 39.2%). The first vignette concerned vancomycin for adult patients infected with methicillin-resistant *Staphylococcus aureus* (MRSA) (n = 90 responses). Trough level target ranges were 15–20 mg/L for 45.6% of respondents, 10–20 mg/L for 35.6% and 10–15 mg/L for 11.1%. Six respondents (6.7%) indicated that they targeted the AUC_0-24_ range of 400–600 mg·h/L rather than trough concentrations (Supplementary Table S2). The second vignette was for voriconazole in adults (*n* = 42 responses). Trough level target ranges were 1–5.5 mg/L for 54.8% of respondents, and 2–4 mg/L for 28.6% as the range in critically ill patients (Supplementary Table S2). 15–20 mg/L for vancomycin and 1.5–5 mg/L for voriconazole were the most often included by the target range (Figure 3). The target range of these two drugs mainly refers to the guidelines (61.1 and 73.8%, respectively). Respondents indicated established critical values were implemented for vancomycin and voriconazole as a hard upper limit (56.7% and 64.3%, respectively). TDM reports included a recommendation at the institutions of 72.7% of the respondents, with the same percentage reporting implementation of ‘active TDM’ in the last 2 weeks (Table 5). 41.4% reported that the number of interventions exceeded half of the total TDM services of the hospital.

**TABLE 5:** Clinical vignettes for interpretation^a^

	Vancomycin (N = 90, 90.0%)		Voriconazole (N = 42, 42.4%)		
Primary reference of target	n	%	n	%
Guideline	55	61.1	31	73.8
Handbook or manual	21	23.3	1	2.4
Expert Consensus Document	12	13.3	8	19.0
Articles	2	2.2	2	4.8
Ideal target				
Trough concentration	76	84.3	42	100
AUC_0-24 _	27	30.0	0	0
AUC_0-24_/MIC	27	31.1	0	0
Peak concentration	1	1.1	0	0
The next round of ordering TDM				
Yes, regularly, even if initially within the targeted range	34	37.8	12	28.6
Yes, but only when not achieving the therapeutic target or after adjustment	50	55.6	25	59.5
No	6	6.7	5	11.9
The primary regimen once TDM results available				
TDM result + clinical effect^b^	20	22.2	17	40.5
TDM result + PD	19	21.1	6	14.3
TDM result + PK	14	15.6	4	9.5
TDM result + software	6	6.7	0	0
A combination of above	18	20.0	5	11.9
Only TDM result	11	12.2	10	23.8
No adjustments	2	2.2	0	0
How is the MIC value obtained generally?				
Microbiology laboratory measurement	72	80.0	28	66.7
A specific guideline /handbook / manual / article	9	10.0	3	7.1
Clinicians’ empirical judgment	1	1.1	1	2.4
No MIC value or unclear	8	8.9	10	23.8
The strategy once resistance (e.g., MIC>1 mg/L)				
Take AUC_0-24_ as a consideration	41	45.6	—^c^	—
Just change drug	31	34.4	—	—
Just increase dosage	8	8.9	—	—
No change	6	6.7	—	—
No MIC value, depending on patients’ response or referral	4	4.4	—	—
A critical value set to initiate a rapid response	51	56.7	27	64.3
No	25	27.8	12	28.6
Unclear	14	15.6	3	7.1
Intervention provided to clinicians	N		%	
Active (before clinician consultation)	72		72.7	
Passive (after clinician consultation)	27		27.3	
Intervention based on TDM (in the last 2 weeks)				
<50% of the total service volume of TDM	41		41.4	
≥50% of the total service volume of TDM	41		41.4	
Unclear	17		17.2	
Interpretation or intervention suggestions shown on TDM report	72		72.7	

a A total of 99 respondents participated in TDM, result.

b “+” means “combining with”.

c “—” means not applicable.

AUC, area under the curve; MIC, minimum inhibitory concentration; TDM, therapeutic drug monitoring; PK, pharmacokinetics; PD, pharmacodynamics.

### The turnaround time of TDM services

**FIGURE 4:**
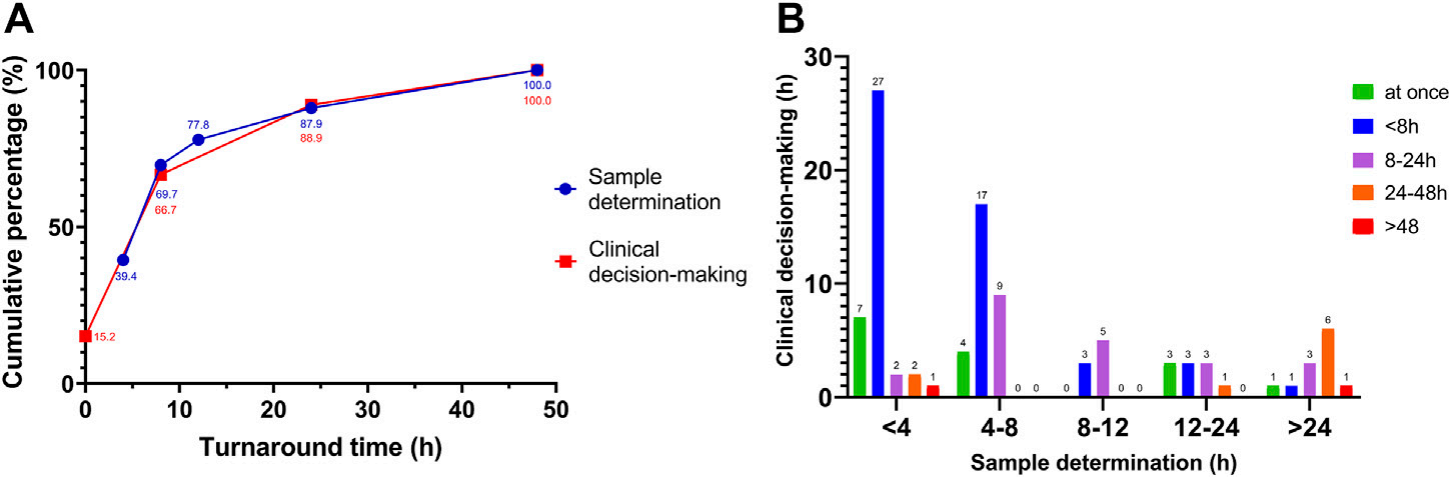
The efficacy of TDM services in sample determination and clinical decision-making. **(A)** the cumulative percentage of these two processes completed over time (150 and 99 responses, respectively); **(B)** the distribution of the 99 responses. The sample determination means from sampling to TDM result; clinical decision-making means from TDM result to clinical response.

The assay result and clinical decision-making can be completed within 24 h in most hospitals (87.9% and 88.9% respectively; Figure 4A). For assay results available within 4 h after sampling, clinical decisions can be made immediately (7 of 39, 17.9%) or within the next 8 h (27 of 39, 69.2%; Figure 4B). As the turnaround time for assay results increased, the proportion of respondents able to make a clinical decision within the next 8 h decreased. For results available after 24 h, two of 12 respondents (16.7%) were able to make a decision within the following 8 h, and six of 12 (50%) within 24–48 h (Figure 4B). In addition, there is a significantly moderate correlation between these two turnaround times, according to the result of nonparametric Spearman’s rank correlation coefficient (the value is 0.373, *p* < 0.01). Furtherly, there was significantly linear correlation between them (the value of χ2 is 16.082, *p* < 0.01).

### The barriers and challenges for TDM

‘Lack of funding or equipment’ was the primary barrier for implementing TDM (71.1% of 150), followed by ‘lack of interest or cooperation from clinicians’ (47.0%) and ‘lack of TDM expertise’ (42.3%; Suplementary Table S3). In contrast, patient cooperation was not considered a barrier (14.8%). Respondents that did not have a TDM service at their institution (18 of 29%, 62.1%; Supplementary Figure S2A) indicated ‘lack of experience’ as the second barrier. Lack of expertise was the primary barrier for using precision dosing software (50.5% of 99). For institutions that do not use dose optimisation software (78% of 99%, 78.8%), the primary barrier was insufficient experience (43 of 78%, 55.1%); followed by lack of availability of minimum inhibitory concentration (MIC) determination (30%, 38.5%) and drug concentration (24%, 30.8%; Supplementary Figure S2B). When dose prediction software was available, respondents expressed concerns with validity of results, insufficient experience with interpretation and software application, and lack of assays for drugs of interest (Supplementary Figure S2B).

## Discussion

To our knowledge, this is the first report concerning TDM practices involving a range of anti-infectives in several Asian countries. Previous studies have been conducted in individual countries, have focused on specific anti-infective classes or have focused on applications in intensive care only (Ohnuma et al., 2018; Choi et al., 2019; Hamada et al., 2020; Liu et al., 2021; Zhang et al., 2021).

We found anti-infective TDM practices varied in terms of drugs involved, sample types, bioanalytical approaches and degree of clinical interpretation of results. TDM is common for drugs with available assays, including vancomycin and aminoglycosides, and TDM of *β*-lactam antibiotics is recommended in international guidelines (Richter et al., 2022), however there are emerging TDM candidates, including fluoroquinolones and linezolid (Roberts et al., 2012; Abdul-Aziz et al., 2020; Sandaradura et al., 2021).

Tuberculosis is of high incidence in the Asia region (World Health Organization, 2021). Efficacious, safe and economical medication treatment is always a priority, in which TDM plays an important role in personalise *tuberculosis* treatment and comparing with individual MICs to explore pharmacokinetics/pharmacodynamics indices. As the most important choices for multidrug-resistant *tuberculosis*, amikacin and linezolid are quantified by immunoassays and LC-MS, respectively (van Altena et al., 2017; Bolhuis et al., 2018).

In this sample, clinicians most frequently ordered TDM and interpretation of TDM was most commonly performed by pharmacists. Although anti-infective TDM was not performed at institutions for 15.3% of all respondents and for almost half of Indian respondents, as well as a limited variety of anti-infectives in Malaysia, their involvement in the survey, especially clinical pharmacologists and academic professionals, suggested significant interest.

In the section of clinical vignettes, we asked questions concerning vancomycin and voriconazole as common TDM candidate drugs with well-established ranges to reflect the anti-infectives TDM clinical interpretation and decision-making based on TDM results more specifically. We found most hospitals targeted range reference followed established guidelines, similar to the result in Australian hospitals (He et al., 2020; Imani et al., 2020). For countries and institutions that have not yet formulated the target, it could be preliminarily reference to 15–20 mg/L or AUC_0-24_ (400–600 mg·h/L) for vancomycin in adults infected with MRSA and 1–5.5 mg/L or 1.5–5 mg/L for voriconazole in adults. Individual differences in patients need to be noted. The target concentration range of voriconazole in critically ill patients may be reduced to 2–4 mg/L clinically, considering the clinical efficacy and safety. It confirms the clinical utility and rational application of TDM, although which is not clearly stated in the guidelines.

The critical values of drug concentration have been established in more than half of these hospitals. But so far, there was little reference of critical values even in the drug guidelines. Significantly, it was first published as early as 1982, which is a specific value of supratherapeutic concentration (e.g. 30 ml/L for vancomycin), to attract great attention to abnormally high exposure and encourage rapid clinical response to prevent the serious adverse drug events (Patton and Borshoff, 2018; Medical Laboratory Observer, 2022). So advocating attention to critical values is still of clinical significance.

We found the efficiency of TDM service can be ensure in most hospitals. The significantly linear correlation between the turnaround times of assay result and clinical decision-making suggested that the more efficient the sampling and assay are, the faster the TDM results can be applied to the follow-up adjustment by clinicians, which may be due to the surrounding concerns for TDM service and the daily schedule of clinicians in the real world. Whilst the role of many possible factors deserves further exploring to make TDM standard of care for anti-infective therapy, including Standard Operating Procedure compliance of sampling and determination, the time lag between serum sample and result, the development of point of care testing, and additional studies to standardize the approach for both reactive and proactive TDM (Strohbehn et al., 2018; Sandaradura et al., 2021). Optimization assay also plays a positive role in the efficient TDM practice and should been encouraged, which will promote clinicians to work based on the guidelines rather than the availability of technology and supplies, especially in most developing countries in Asia (Decosterd et al., 2020; Fuentes et al., 2021).

It was reassuring that the practice of TDM is expanding, accompanied by the emphasis on the innovation of instruments and methods (Dhaese et al., 2020). Pharmacokinetics and pharmacodynamics of anti-infectives based on TDM are increasingly acknowledged as a key component for optimal treatment, the prevention of a slow response to treatment, acquired drug resistance, and adverse drug effects. Therefore, further stimulation to initiate TDM is required (Sturkenboom et al., 2021). Lack of funding and equipment, lack of cooperation from clinicians, and lack of TDM expertise are considered the major barriers to TDM practice. Removing all identified barriers may begin with concerted efforts to refine TDM programmes at an institutional and national level, including establishing and implementation of standardized TDM interpretation scheme and guidelines, increasing the availability of standardized and accurate TDM assays, and carrying out professional knowledge training and exchange in ordering, sampling and interpretation for clinicians, pharmacists and nurses. TDM not only represents concentration measured, but also should be reflected in follow-up clinical decision-making. The clinical interpretation of TDM results by trained professionals plays a key role, which helps to tailor antimicrobial therapy, especially for special populations with different pathophysiology and pathogen’s susceptibility, including critically ill patients, the elderly, obesity, pediatric patients, and patients with renal failure (Gatti et al., 2022; Schmid et al., 2022). With the development of technology, more and more dose optimization software based on TDM was developed. Our survey revealed that only 21.2% institutions are using software to overcome some challenges, less than the Australian ones (51%) (Imani et al., 2020). And the challenges reported suggested that in addition to training in the use of dose optimisation software, developing and improving high-quality software is also important (Wicha et al., 2021).

There are some limitations to this work. Although every effort was made for widespread reach, respondents were primarily from China, Malaysia and India, meaning that results reflect essentially TDM practice of anti-infective agents in these three countries, and that in the other countries, TDM of anti-infective agents needs to be implemented, needing deeper and more widespread studies in further. As for any self-reported data, may or may not representing the wider practice in that institution or the local guidelines, there is also a risk of social desirability bias or reporting bias (Delannoy et al., 2019). It is therefore possible that our results present a more optimistic picture than in reality. Although clinical vignettes are widely used to evaluate clinical practices in the real-world (Das et al., 2015), responses may not reflect the complexities and nuances of clinical decision-making at the bedside. Although our study involved critically ill patients in the clinical vignettes, the lack of characteristics of the patient population managed by survey responders means that the results can not accurately reflect the different responses taken to different patient profiles, which is very meaningful for individual application of TDM service and needs further surveys. Despite the above limitations and preliminary understanding, we believe that these data are important to emphasize the initiation, efficiency, accuracy of intervention, and process improvement of anti-infective TDM practices.

## Conclusion

The results point to TDM for some anti-infectives (vancomycin, aminoglycoside and voriconazole) is common across countries in Asia. Whereas TDM practices for other anti-infectives varies. TDM practice is being promoting and considerable in Asia, although it may not yet be widely available. A coordinated effort TDM procedure is needed to refine TDM programmes at an institutional and national level, including developing and implementation available and efficient assays, standardized interpretation scheme and guidelines, professional knowledge training and exchange in ordering and interpretation for clinicians and pharmacists, and the application of dose optimisation software. There is an urgency for well conducted research that will allow the understanding the phenomenon and seek the solution related to this topic more deeply and accurately.

## Data Availability

The raw data supporting the conclusions of this article will be made available by the authors, without undue reservation.
