# Supplementary material for: Therapeutic drug monitoring practices of anti-infectives: An Asia-wide cross-sectional survey
Source: Front Pharmacol. 2022 Oct 10;13:992354. doi: 10.3389/fphar.2022.992354 (PMC9589087; doi:10.3389/fphar.2022.992354)
Supplement: Supplementary file 1 [file DataSheet1.docx]

Supplementary Material

# Supplementary Figures and Tables

## Supplementary Figures


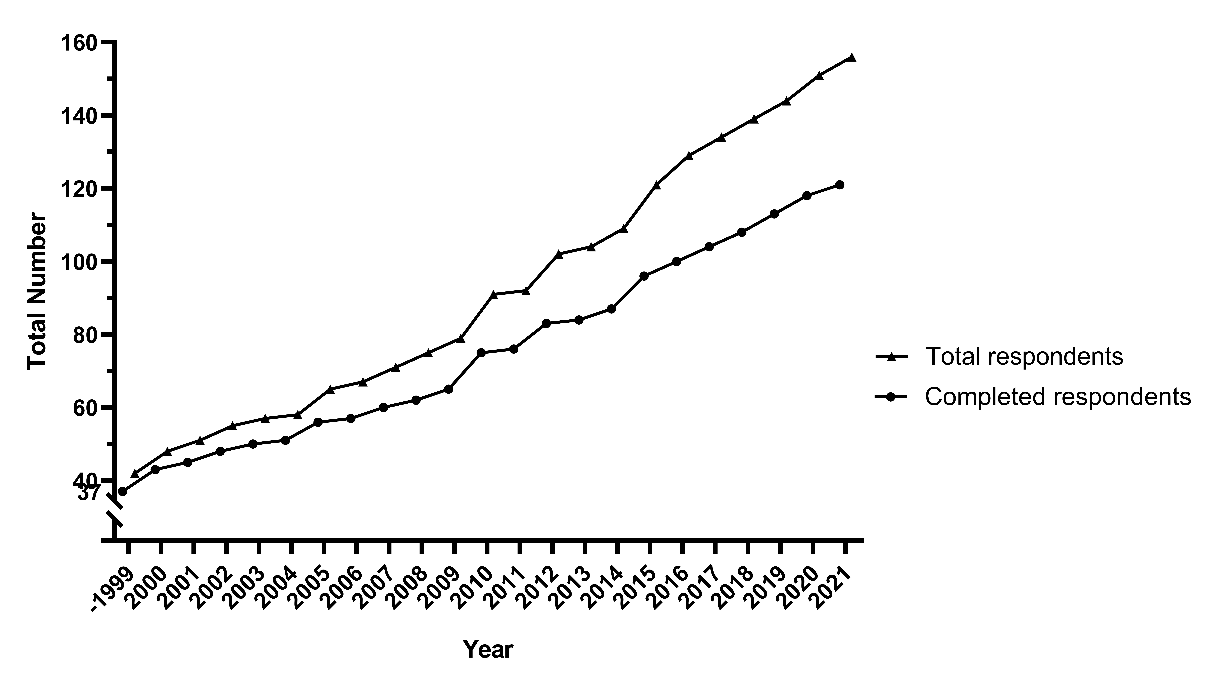


**Supplementary Figure 1.** The distribution of TDM service commencement over time for the Asian institutions (156 of 205 total qualified respondents including incomplete ones, and 121 of 150 completed ones, respectively).


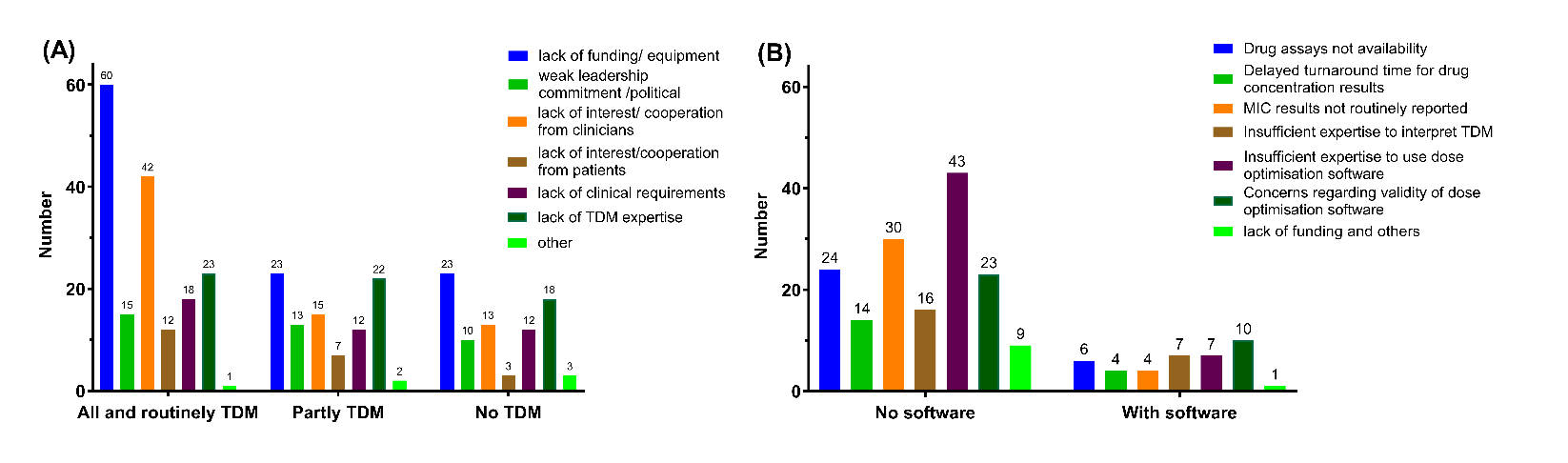


**Supplementary Figure 2.** The barriers faced by institutions in different current situations.

## Supplementary tables

**Supplementary Table 1.** The TDM available anti-infective agents of China, Malaysia and India.

|  | **China (N=54)** | **Malaysia (N=41)** | **India (N=31)** |
| --- | --- | --- | --- |
| **None of any anti-infectives** **n (%)** | 2 (3.7) | 1 (2.4) | 15 (48.4) |
| **Antibacterial drugs** |  |  |  |
| None | 4 (7.4) | 1 (2.4) | 16 (51.6) |
| Glycopeptides (Vancomycin, Norvancomycin, Teicoplanin, etc.) | 44 (81.5) | 39 (95.1) | 10 (32.3) |
| Aminoglycosides (Gentamicin, Amikacin, Tobramycin, etc.) | 16 (29.6) | 38 (92.7) | 3 (9.7) |
| β-lactams (including the carbapenems such as Meropenem.) | 25 (46.3) | 1 (2.4) | 5 (16.1) |
| Oxazolidinones (e.g., linezolid) | 19 (35.2) | 0 (0.0) | 2 (6.5) |
| Polypeptide antibiotic (e.g., polymyxin B) | 14 (25.9) | 0 (0.0) | 2 (6.5) |
| Quinolones (e.g., moxifloxacin, ciprofloxacin) | 4 (7.4) | 0 (0.0) | 1 (3.2) |
| Sulfonamides (e.g., Sulfamethoxazole) | 3 (5.6) | 0 (0.0) | 1 (3.2) |
| Tigecycline | 2 (3.7) | 0 (0.0) | 0 (0.0) |
| Fosfomycin | 0 (0.0) | 0 (0.0) | 0 (0.0) |
| Unclear | 2 (3.7) | 1 (2.4) | 3 (9.7) |
| **Antifungal drugs** |  |  |  |
| None | 10 (18.5) | 41 (100.0) | 18 (58.1) |
| Voriconazole | 42 (77.8) | 0 (0.0) | (7 22.6) |
| Posaconazole | 10 (18.5) | 0 (0.0) | (5 16).1 |
| Itraconazole | 9 (16.7) | 0 (0.0) | (1 3.2) |
| Amphotericin B | 4 (7.4) | 0 (0.0) | (6 19.4) |
| Caspofungin | 6 (11.1) | 0 (0.0) | (2 6.5 |
| Flucytosine | 3 (5.6) | 0 (0.0) | 0 (0.0) |
| Isavuconazole | 1 (1.9) | 0 (0.0) | 0 (0.0) |
| Unclear | 0 (0.0) | 0 (0.0) | 0 (0.0) |
| **Anti-tuberculosis drugs** |  |  |  |
| None | 16 (29.6) | 15 (36.6) | 23 (74.2) |
| Isoniazid | 11 (20.4) | 1 (2.4) | 7 (22.6) |
| Rifampicin | 8 (14.8) | 1 (2.4) | 6 (19.4) |
| Pyrazinamide | 5 (9.3) | 1 (2.4) | 4 (12.9) |
| Streptomycin | 2 (3.7) | 2 (4.9) | 3 (9.7) |
| Ethambutols | 3 (5.6) | 0 (0.0) | 3 (9.7) |
| Rifabutin | 4 (7.4) | 0 (0.0) | 0 (0.0) |
| Para-amino salicylic acid | 0 (0.0) | 0 (0.0) | 1 (3.2) |
| Kanamycin | 3 (5.6) | 0 (0.0) | 1 (3.2) |
| Linezolid | 16 (29.6) | 0 (0.0) | 2 (6.5) |
| Ethionamide | 0 (0.0) | 0 (0.0) | 1 (3.2) |
| Levofloxacin | 6 (11.1) | 1 (2.4) | 1 (3.2) |
| Moxifloxacin | 3 (5.6) | 0 (0.0) | 1 (3.2) |
| Bedaqualine | 1 (1.9) | 0 (0.0) | 1 (3.2) |
| Clofazimine | 0 (0.0) | 0 (0.0) | 1 (3.2) |
| Delamanid | 0 (0.0) | 0 (0.0) | 1 (3.2) |
| Imipenem-cilastatin | 9 (16.7) | 0 (0.0) | 1 (3.2) |
| Amikacin | 13 (24.1) | 26 (63.4) | 1 (3.2) |
| Meropenem | 15 (27.8) | 1 (2.4) | 3 (9.7) |

Supplementary Table 2. Clinical Vignettes for the target range^1^

| **The target trough concentration range for adult patients infected with MRSA** | **N** | | **%** |
| --- | --- | --- | --- |
|  | **Vancomycin (N=90)** | | |
| 15-20 mg/L | 41 | 45.6 | |
| 10-20 mg/L | 32 | 35.6 | |
| 10-15 mg/L | 10 | 11.1 | |
| 5-10 mg/L | 1 | 1.1 | |
| AUC_0-24_ (400-600 mg·h/L) | 6 | 6.7 | |
| **The target trough concentration range for adult patients (mg/L)** | **Voriconazole (N=42)** | | |
| 0.5-5 | 11 | 26.2 | |
| 1-5.5 | 23 | 54.8 | |
| 1.5-6 | 3 | 7.1 | |
| 2-6 | 2 | 4.8 | |
| 1-4 | 1 | 1.1 | |
| 1-5 | 1 | 1.1 | |
| 1.5-5.5 | 1 | 1.1 | |
| **Pursuing a smaller target range, considering clinical efficacy and safety in critically ill patients** | **Voriconazole (N=42)** | | |
| Yes, 2-4 mg/L | 12 | | 28.6 |
| No | 30 | | 71.4 |

^1^ A total of 99 respondents participated in TDM result.

MRAS, Methicillin-resistant Staphylococcus aureus

Supplementary Table 3. The barriers and challenges for TDM

|  | N | % |
| --- | --- | --- |
| **For TDM implement** | N=150 |  |
| Lack of funding or equipment | 106 | 71.1 |
| Weak leadership commitment or political | 37 | 24.8 |
| Lack of interest or cooperation from clinicians | 70 | 47.0 |
| Lack of clinical requirements | 42 | 28.2 |
| Lack of TDM expertise | 63 | 42.3 |
| Lack of interest or co-operation from patients | 22 | 14.8 |
| Other | 6 | 4 |
| **Why are no interpretation or intervention suggestions shown on TDM report?** | N=27^1^ |  |
| No needed because there is already a targeted concentration range | 7 | 25.9 |
| Lack of required clinically | 7 | 25.9 |
| Lack of knowledge | 7 | 25.9 |
| Lack of time | 6 | 22.2 |
| **For the implement of intervention practice** | N=99 |  |
| No promotion of authority or lack of organization communication and coordination | 32 | 32.3 |
| Lack of relevant knowledge for interpreter | 31 | 31.3 |
| Lack of requirement, trust, or support of clinicians | 27 | 27.3 |
| Other | 9 | 9.1 |
| **For performing TDM dose optimisation software** | N=99 |  |
| Drug assays not availability | 30 | 30.3 |
| Delayed turnaround time for drug concentration results | 18 | 18.2 |
| MIC results not routinely reported | 34 | 34.3 |
| Insufficient expertise to interpret TDM | 23 | 23.2 |
| Insufficient expertise to use dose optimisation software | 50 | 50.5 |
| Concerns regarding validity of dose optimisation software | 33 | 33.3 |
| Other | 10 | 10.1 |

^1^ A total of 27 respondents are eligible and answered the question.
